# Underwater small target detection under YOLOv8-LA model

**DOI:** 10.1038/s41598-024-66950-w

**Published:** 2024-07-12

**Authors:** Shenming Qu, Can Cui, Jiale Duan, Yongyong Lu, Zilong Pang

**Affiliations:** School of Software, Henan University, Kaifeng, 475004 Henan China

**Keywords:** Underwater image processing, Small target detection, PConv, FasterNet, YOLOv8, Neural network, Computer science, Software

## Abstract

In the realm of marine environmental engineering, the swift and accurate detection of underwater targets is of considerable significance. Recently, methods based on Convolutional Neural Networks (CNN) have been applied to enhance the detection of such targets. However, deep neural networks usually require a large number of parameters, resulting in slow processing speed. Meanwhile, existing methods present challenges in accurate detection when facing small and densely arranged underwater targets. To address these issues, we propose a new neural network model, YOLOv8-LA, for improving the detection performance of underwater targets. First, we design a Lightweight Efficient Partial Convolution (LEPC) module to optimize spatial feature extraction by selectively processing input channels to improve efficiency and significantly reduce redundant computation and storage requirements. Second, we developed the AP-FasterNet architecture for small targets that are commonly found in underwater datasets. By integrating depth-separable convolutions with different expansion rates into FasterNet, AP-FasterNet enhances the model’s ability to capture detailed features of small targets. Finally, we integrate the lightweight and efficient content-aware reorganization (CARAFE) up-sampling operation into YOLOv8 to enhance the model performance by aggregating contextual information over a large perceptual field and mitigating information loss during up-sampling.Evaluation results on the URPC2021 dataset show that the YOLOv8-LA model achieves 84.7% mean accuracy (mAP) on a single Nvidia GeForce RTX 3090 and operates at 189.3 frames per second (FPS), demonstrating that it outperforms existing state-of-the-art methods in terms of performance. This result demonstrates the model’s ability to ensure high detection accuracy while maintaining real-time processing capabilities.

## Introduction

Underwater target detection technology is pivotal for identifying and monitoring targets within environments characterized by limited visibility. It is extensively employed in diverse fields including marine ecosystem research, underwater navigation, and the estimation of marine biological populations. Additionally, this technology plays a critical role in deep-sea fisheries and the detection of submerged explosives, thereby significantly enhancing the exploration and sustainable exploitation of marine resources^1–4^. Given that current marine operations predominantly rely on human involvement, which incurs substantial costs due to the labor-intensive nature of tasks such as fishing, the development and implementation of automated detection technologies are of paramount importance. These technologies hold significant potential to assist with marine operations and accelerate research in the field, thus reducing reliance on human labor and fostering advancements in marine studies.

**Figure 1:**
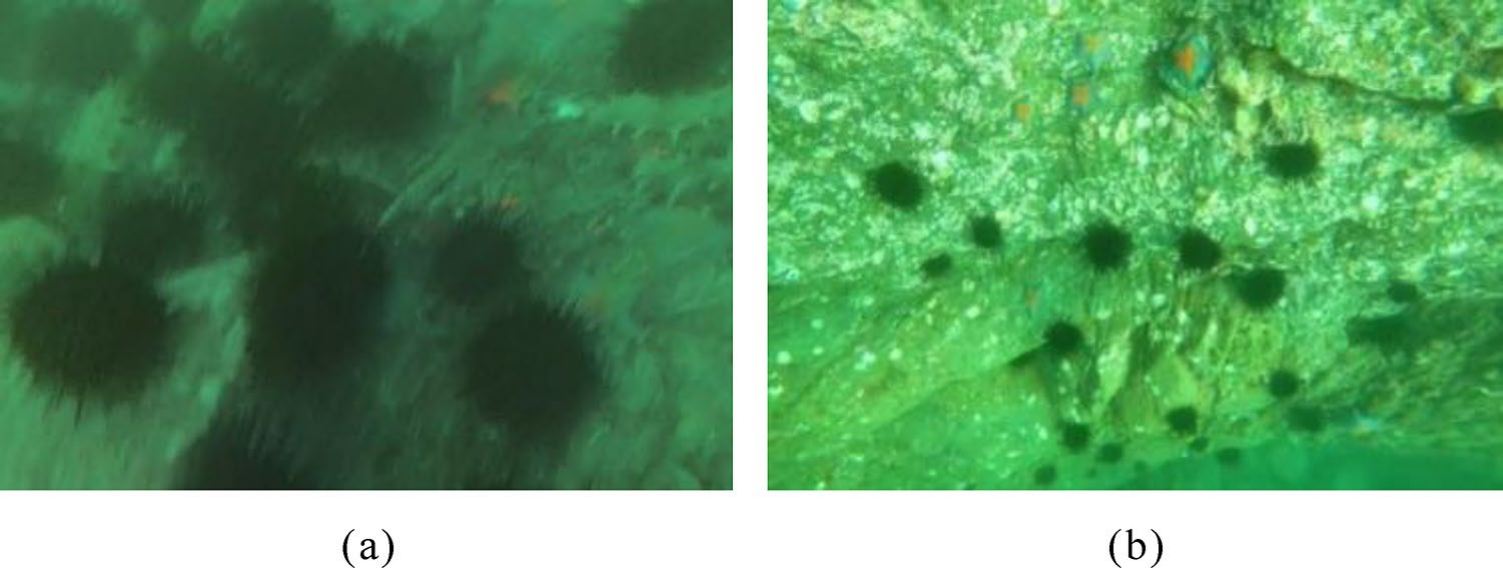
Image displaying small, densely packed targets within a typical underwater setting, indicating the complexity of detection.

However, the deployment of underwater target detection systems is impeded by a multitude of challenges inherent to marine environments.A primary concern is the dynamic nature of the underwater environment, which requires real-time processing capabilities.Meanwhile, due to the limitations of existing technologies, the embedded platforms usually equipped with Marine robots can only provide extremely limited computing power. This limitation increases the difficulty of developing robust detection algorithms.Additionally,most underwater objects, such as fish schools^5^ and benthic organisms^6^, are typically very small and tend to cluster together in dense distributions due to their natural behavior. In fact, the vast majority of these objects occupy only 0.3 to 1.5% of the image area, which further complicates the detection tasks, as illustrated in Fig. 1. Consequently, underwater target detection remains a particularly challenging area within computer vision, necessitating continual refinement of methodologies to ensure dependable outcomes^7^.

The emergence of deep learning has brought a new light to feature extraction in underwater object detection. Currently, popular target detection algorithms are mainly categorized into two-stage and single-stage methods^8–12^. Two-stage detection methods such as region-based convolutional neural networks (R-CNN) significantly improve the detection accuracy. Fast R-CNN and Faster R-CNN enhanced the efficiency by introducing region proposal networks (RPNs), making real-time object detection feasible^13^. These methods first generate candidate regions and then classify and regress these regions. In contrast, single-stage detectors such as Single-Session Detector (SSD) and You Only Look Once (YOLO) achieve high speed and high accuracy by eliminating region suggestions and performing detection directly on the full map^14–17^. Recent advances have focused on improving detection performance under challenging conditions, such as detecting small targets^18–20^. The Feature Pyramid Network (FPN) proposed by Lin et al. constructs high-level semantic feature maps at all scales by using a top-down architecture with lateral connectivity, and naturally forms a multiscale multi-scalar feature representation during forward propagation by leveraging the intrinsic pyramid structure of CNNs feature representation, which improves the detection accuracy of various target sizes^21^.

In the field of underwater target detection, research based on the two-stage detection framework has made rapid progress. For example, Zeng et al. developed a new framework to robustly detect underwater seafood by integrating a generative adversarial network into a standard Faster R-CNN^22^. However,the method has a complex network structure and a high parametric load. In contrast, the single-stage framework avoids the use of region suggestions and directly extracts hierarchical features to predict the detection results, demonstrating robust real-time processing capabilities. Li et al. proposed an underwater scallop recognition algorithm based on an improved YOLOv5, which achieves fast and accurate scallop detection^23^. Further progress is achieved by integrating new architectures and enhancements. For example, Lei et al. used Swin Transformer in the YOLOv5 framework to improve the detection of blurred underwater images^24^. Similarly, Liu et al. incorporated a CBAM attention module in YOLOv5 and performed image enhancement through a global histogram algorithm to improve underwater image quality^25^. In addition, Ji et al. proposed a joint image enhancement and super-resolution technique for underwater target detection combined with a multi-head fuzzy fusion network to capture contextual information^26^.

Despite these innovations, the complexity of the network architecture and image enhancement strategies significantly hinders the efficiency of the training and inference process, as well as increases the risk of overfitting. Moreover, in scenarios involving smaller targets, these approaches tend to extract redundant noisy features, significantly reducing performance. Meanwhile, existing multiscale feature fusion techniques tend to rely on fixed linear feature aggregation methods that ignore contextual information, thus limiting their effectiveness in complex scenarios.

To further enhance the extraction of complex features from underwater targets, we propose a novel detection model featuring two key innovations: the LEPC module and the AP-FasterNet module. The LEPC module replaced part of the C2F module in YOLOv8, employing partial convolution on select input channels to reduce the parameter count and boost computational speed, while enabling multi-stage parameter sharing to streamline parameter use across different convolution stages. Meanwhile,the AP-FasterNet module draws on the idea of Feature Pyramid Networks (FPN) to enhance the detection of small targets through efficient fusion of local and global information. Furthermore, the addition of residual connections and grouped convolutions enhances inter-layer feature complementarity and network stability, thereby improving the model’s adaptability to complex scenarios.

**Figure 2:**
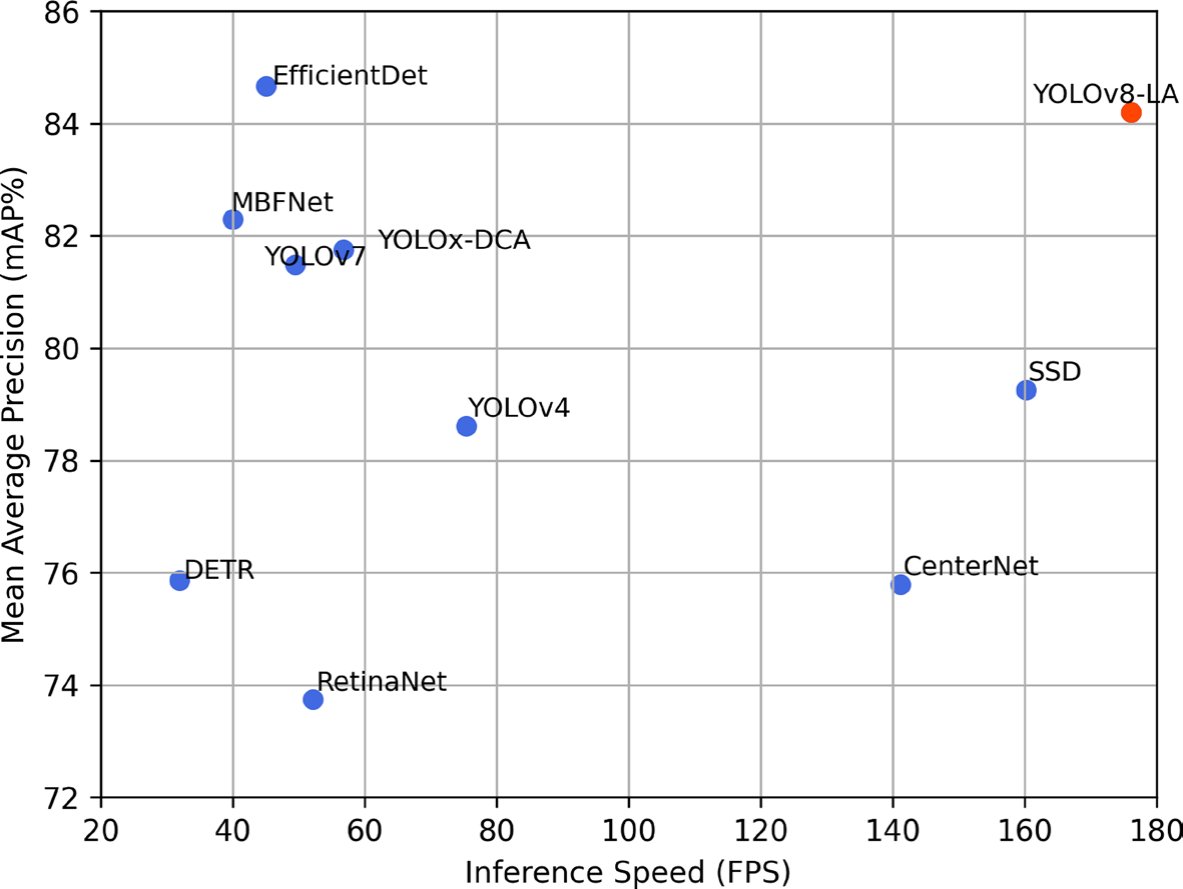
Performance comparison of different underwater target detection algorithms on the Zhanjiang competition dataset.

As shown in Fig. 2, the YOLOv8-LA model, equipped with these innovations, achieves superior detection accuracy over mainstream methods while maintaining real-time performance.

The contribution of this article is summarized as follows:

1. We proposed the LEPC module to replace the C2F module in YOLOv8, reducing parameter count and enhancing computational speed through selective convolution.
2. Designed the AP-FasterNet module, replacing the feature extraction module of YOLOv8. Enhanced the accuracy of small object detection and improved network stability.
3. The upsampling module CARAFE was introduced, reducing computational complexity while effectively extracting rich semantic features.

The remainder of the paper is structured as follows: Section “Method” provides an overview of underwater target detection methodologies. Section “LEPC module” explores the operational mechanisms and design principles of LEPC and AP-FasterNet, elaborating on their integration with the YOLOv8 architecture and evaluating their effectiveness. Section “Experimental results and analysis” outlines the experimental setup, presents detection results, and compares these with the advanced neural network. Additionally, the enhancements facilitated by these two modules are discussed through ablation experiments. Finally, section “Conclusion” Concludes the paper and suggests directions for future work.

## Method

### YOLOv8

To ensure adequate real-time detection capabilities, we selected the YOLO8 framework. While YOLOv8 has demonstrated exceptional performance in standard scenarios, it exhibits certain limitations in special and complex application scenarios, such as intensive target and small target detection. Consequently, this study embarked on a comprehensive exploration and optimization of the YOLOv8 network architecture to better accommodate underwater target detection characteristics.

The YOLOv8 architecture is structured into several components: the input layer, backbone network, neck, head, and output layer. The backbone network, fundamental to the architecture, is comprised of a series of convolutional layers that extract features from the input image. This network integrates the C2f module, which includes a cross-stage partial bottleneck with two convolutions, enhancing the integration of advanced functionality and contextual information. Additionally, the backbone utilizes the Spatial Pyramid Pooling (SPP) module, which effectively captures object features across multiple scales, enriching the feature extraction process. The neck of the network consists of additional convolutional layers that refine the feature maps produced by the backbone, ensuring a richer representation of the input data. The head of the network comprises several convolutional layers that generate bounding box predictions and class probabilities for each grid cell within the output feature map, facilitating accurate object localization and classification. Finally, the output layer of YOLOv8 generates the final object detection predictions, which include class labels, bounding box coordinates, and confidence scores. This ensures precise and reliable outcomes in object detection across varied conditions^16^.

**Figure 3:**
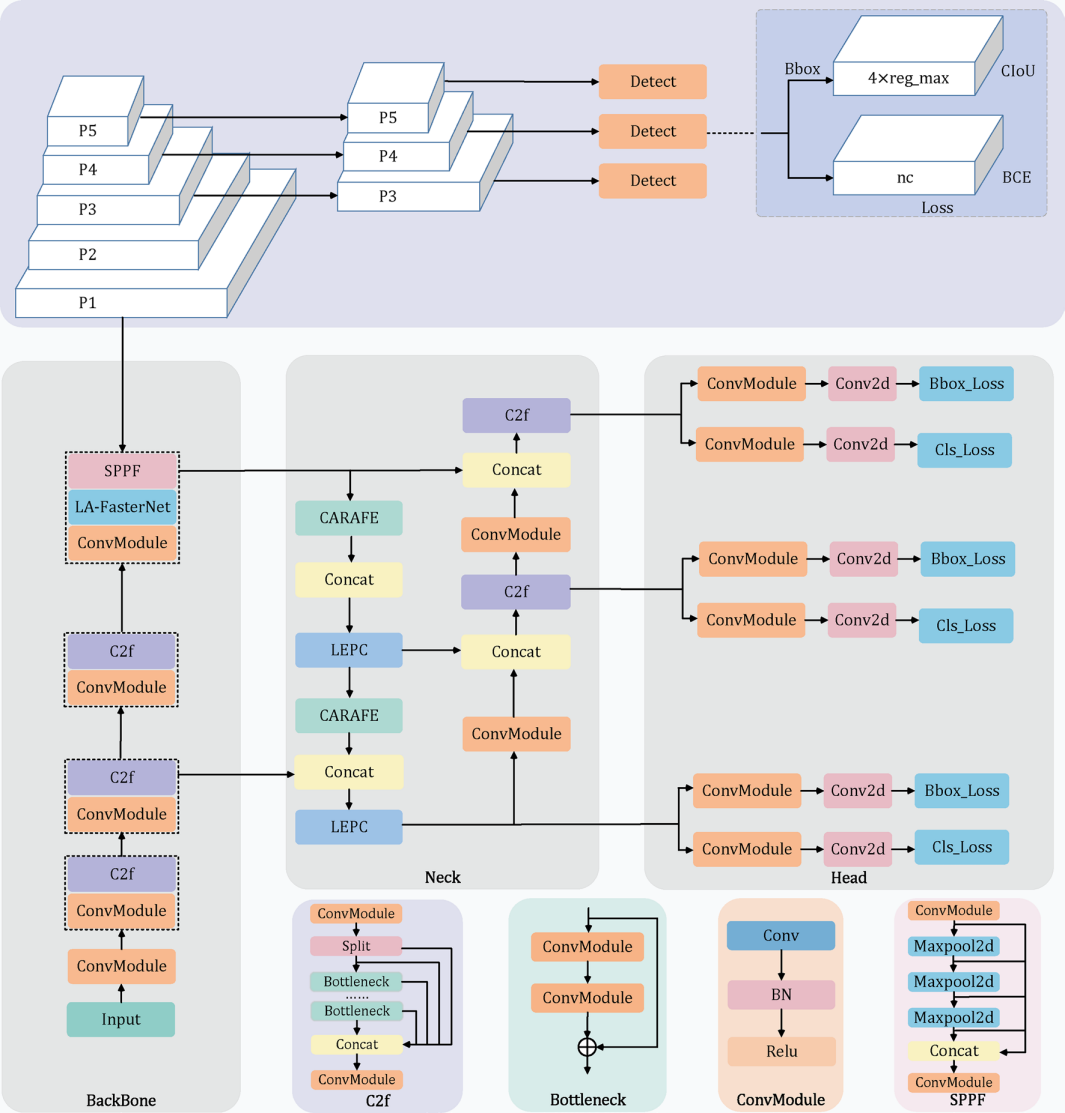
YOLOv8-LA network model.

While YOLOv8 has improved feature integration and contextual information processing with its C2f modules, it still faces challenges in detecting small and densely packed targets, particularly in complex underwater environments. Additionally, while the feature fusion in the backbone network has its advantages, it suffers from an excess of parameters and inefficiencies. To address these issues, the proposed YOLOv8-LA incorporates the efficient AP-FasterNet as its backbone, replacing the original network structure. In the neck section, the LEPC module is used to enhance feature extraction. Moreover, the CARAFE module replaces traditional upsampling to minimize information loss and improve feature representation. The architectural design of YOLOv8-LA is depicted in Fig. 3.

### LEPC module

In the field of underwater image detection, reducing the number of parameters is crucial for improving detection speed. The substantial parameter count in the C2f module of YOLOv8 stems from its complex convolutional operations and repetitive Bottleneck module design. While this design enhances feature processing capabilities, it also significantly increases computational overhead, particularly in resource-constrained environments, thereby reducing inference speed.

**Figure 4:**
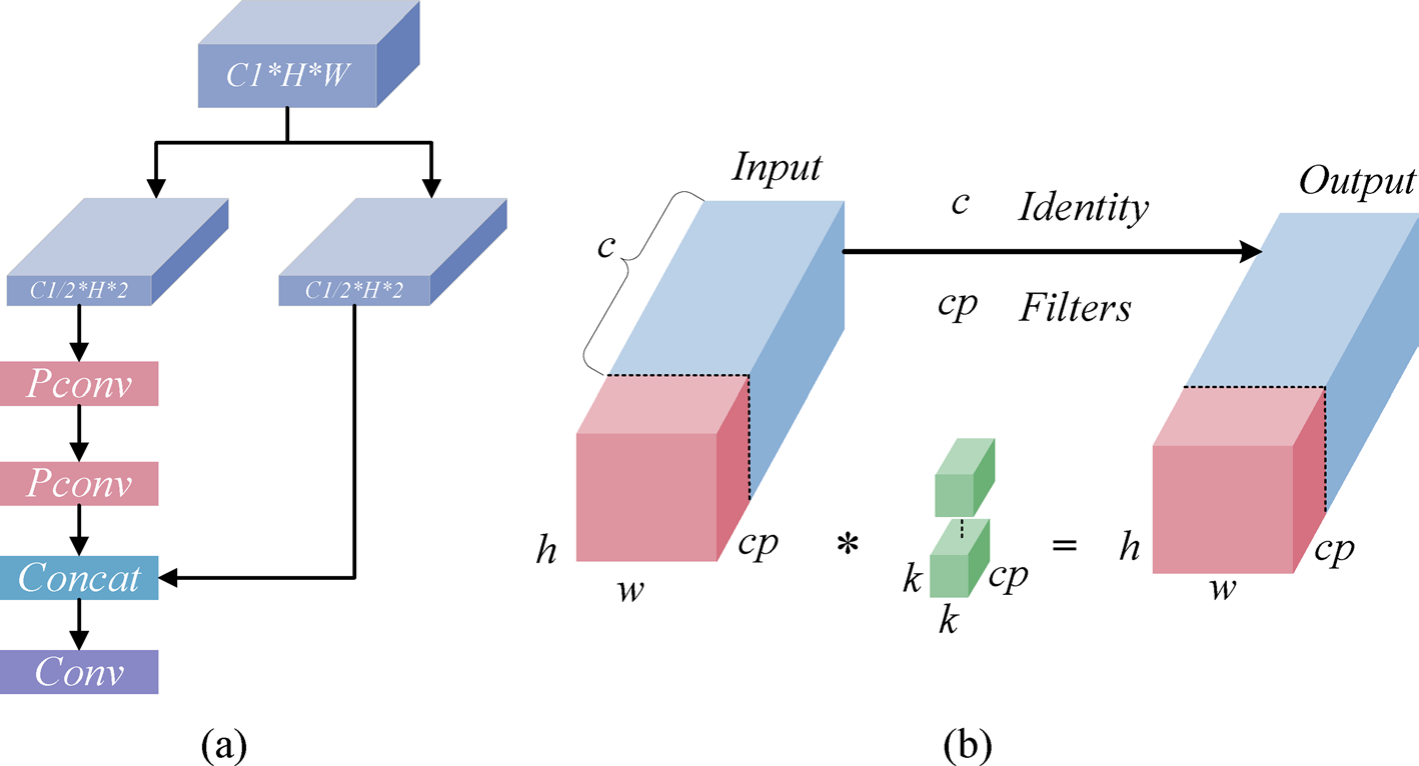
Schematic diagram of the LEPC module structure. (**a**) LEPC module. (**b**) PConv, *representing convolution operation.

To effectively address the issue of a large number of parameters, we proposed the Lightweight and Efficient Partial Convolution (LEPC) module. This module is designed to reduce the parameter count while simultaneously enhancing feature processing efficiency. The architectural design of LEPC is demonstrated in Fig. 4a. The LEPC module operates on the foundational principles of partial convolution, utilizing a dual-stage approach. Each stage selectively processes only a subset of the input channels by dividing them into smaller groups and applying convolution to only part of these groups at a time, leaving the rest unaltered. This method, akin to the partial convolution illustrated in the provided PConv analysis, significantly reduces the FLOPs required for processing.

Figure 4b elucidates the operational mechanism of PConv,which represents the input feature map as a three-dimensional tensor with dimensions denoted by $h\times w\times c$, where h, w, and *c* represent the height, width, and number of channels of the feature map, respectively. PConv employs a convolutional filter of size $k \times k \times c_{p}$ to process the first quarter of input features, while retaining the last three-quarters of input features through residual connections to ensure smooth transmission of information in the network. The complexity of a model is frequently assessed in terms of floating point operations (FLOPs)^17^, and the FLOPs of PConv are $h\times w\times k^{2} \times c_{p}^{2}$. As shown in the figure, since $c_{p}$ = c/4 (c represents the size of a regular convolution filter), the FLOPs of PConv are only 1/16 of those of regular convolution.

The C2f module’s parameter calculation, given by $2\times (k\times k\times c\times c)=2c^{2}k^{2}$, uses a traditional convolutional approach where each layer processes the entire feature map using a complete $k\times k\times c\times c$ filter, maintaining a high parameter load.In contrast, the LEPC module utilizes partial convolution to reduce the parameter load innovatively. Each partial convolution within LEPC has its parameters calculated as $(k\times k\times c/4\times c/4)=1/16c^{2}k^{2}$. This approach, similar to PConv, significantly decreases the number of parameters by focusing the convolution on a fraction of the input channels. As a result, LEPC reduces both computational complexity and parameter count, enhancing processing efficiency, particularly in resource-constrained environments.

### AP-FasterNet module

Due to the extremely small size of tiny targets, their representation on the input feature map requires a focus on accurately capturing subtle details.The YOLOv8’s C2f module’s limited receptive field impedes the extraction of these critical features, thereby affecting the precise identification of small targets.

**Figure 5:**
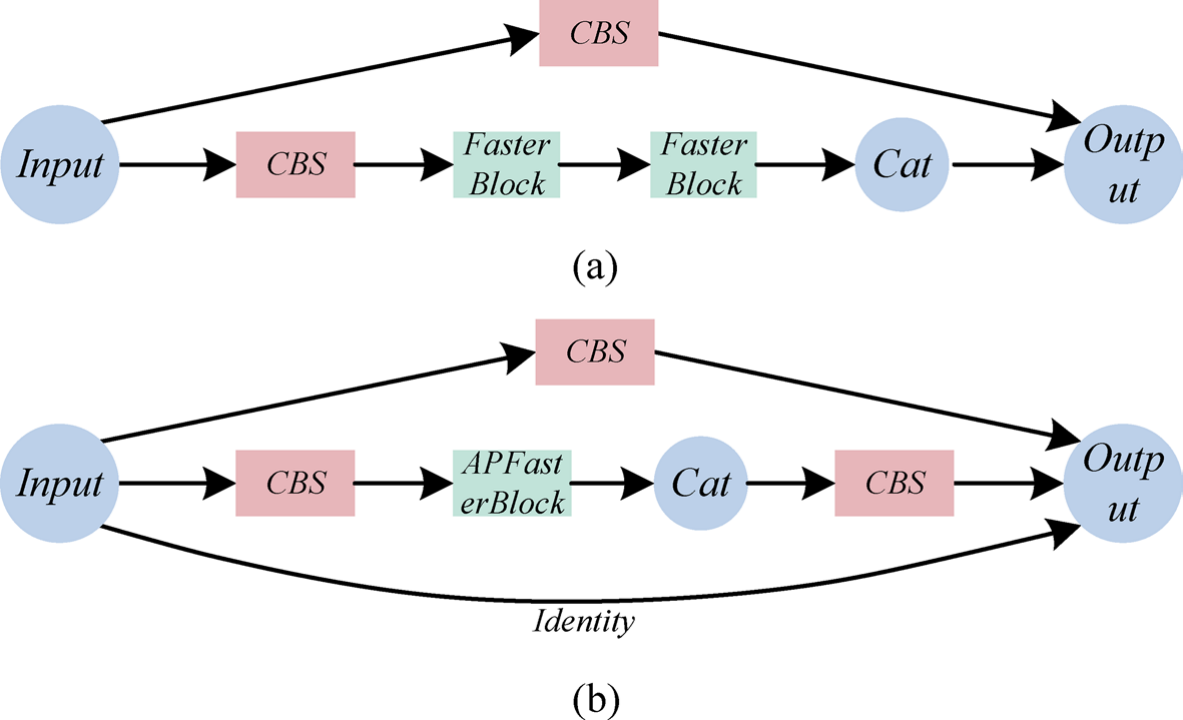
Presents two types of network structures: (**a**) displays the structure of the FasterNet network; (**b**) shows the structure of the AP-FasterNet network. CBS consists of Conv, BN, and SiLU.

**Figure 6:**
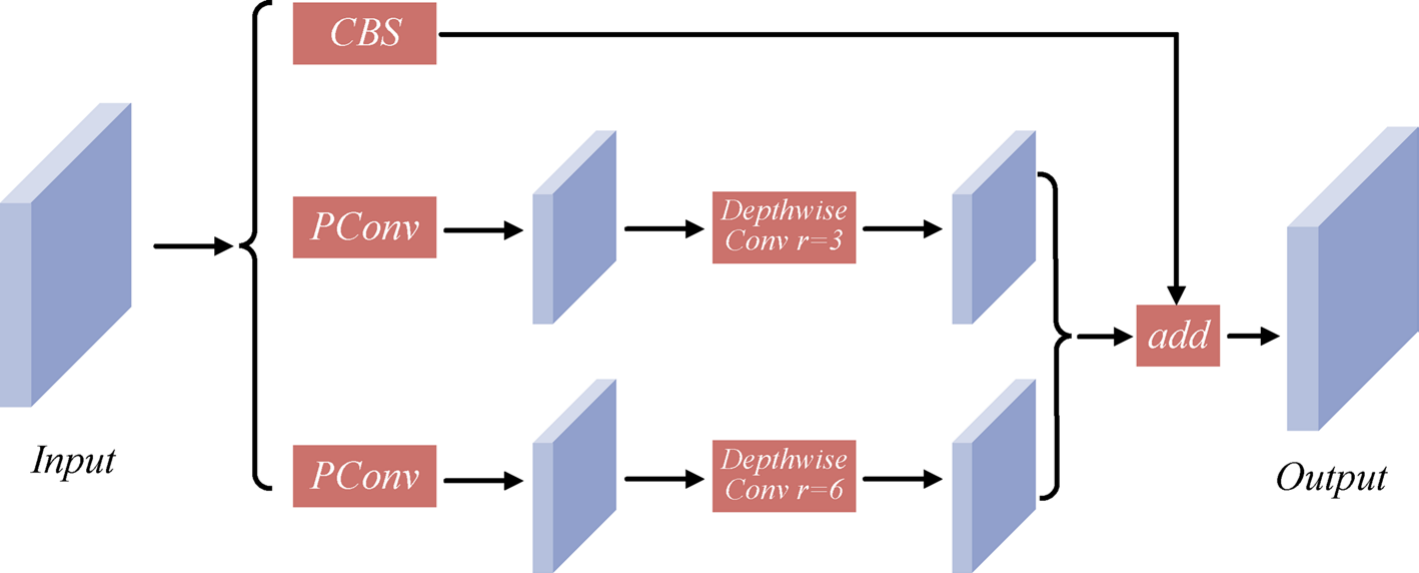
The illustration of the AP-FasterBlock.

In light of this, our study selected FasterNet as the foundational infrastructure and proposed a new network, AP-FasterNet^17^. The overall architecture is shown in Fig. 5. The key innovation of AP-FasterNet is its ability to reduce the number of parameters and improve the detection of small targets. By employing Depthwise Separable Convolutions, which decompose the convolution operation into depthwise and pointwise convolutions, the model significantly reduces the parameter count and computational cost while maintaining effectiveness^27^. Additionally, the Partitioned Convolution divides input channels into processed and untouched segments, enhancing efficiency by reducing redundancy in feature processing and computational load on the system. These strategies minimize the model’s parameter footprint and streamline the computational process without compromising the integrity or accuracy of the model’s output, the AP-FasterBlock architecture is shown in Fig. 6.

The AP-FasterNet module employs dilated convolutions at various rates, expanding the receptive field without increasing the parameter count. This multi-scale context capture is crucial for detecting small and densely packed underwater targets^18,28^. By using different dilation rates, we enlarge the receptive field within the same layer, capturing extensive contextual information essential for precise identification and localization of small targets. Unlike downsampling or strided convolutions, dilated convolutions maintain the spatial resolution of feature maps. This preservation is vital for detecting small targets, as high-resolution feature maps retain detailed information, thus improving detection accuracy. Moreover, the AP-FasterNet module optimizes parameter count and computational complexity. Despite the larger receptive field, the parameter count remains constant, allowing the model to handle extensive contextual information without significantly increasing computational complexity or memory usage. Integrating this module between the encoder and decoder stages balances detail and context, enhancing decoding performance and improving the accuracy of detecting small and densely packed underwater targets.

Meanwhile,the use of Depthwise-Separable expansion convolution within the AP-FasterNet module reduces parameter count and computational cost. The formula definition is given by $y[i,j] = \sum \limits _{m,n} x[i + m \cdot d, j + n \cdot d] \cdot k[m,n]$,Where *y*[*i*, *j*] represents the output feature map at position (*i*, *j*). Here *x* is the input feature map, with $\textbf{x}[i + m \cdot d, j + n \cdot d]$ indicating the input pixel at position $(i + m \cdot d, j + n \cdot d)$. The kernel k contains weights *k*[*m*, *n*] at position (*m*, *n*). The dilation rate *d* determines the spacing between kernel points, effectively expanding the kernel’s receptive field to cover a larger area of the input feature map without increasing parameter count.

Additionally, to address issues such as gradient vanishing and explosion during the detection of small targets, we incorporated residual connections. Residual connection enable input information to span multiple levels through the identity mapping mechanism, promoting rapid information flow within the network^19^. This approach not only boosts the stability and effectiveness of the network but also ensures that the network can learn the main features more deeply during training, maintaining stable gradient flow during both forward and backward propagation.

### CARAFE upsampling

**Figure 7:**
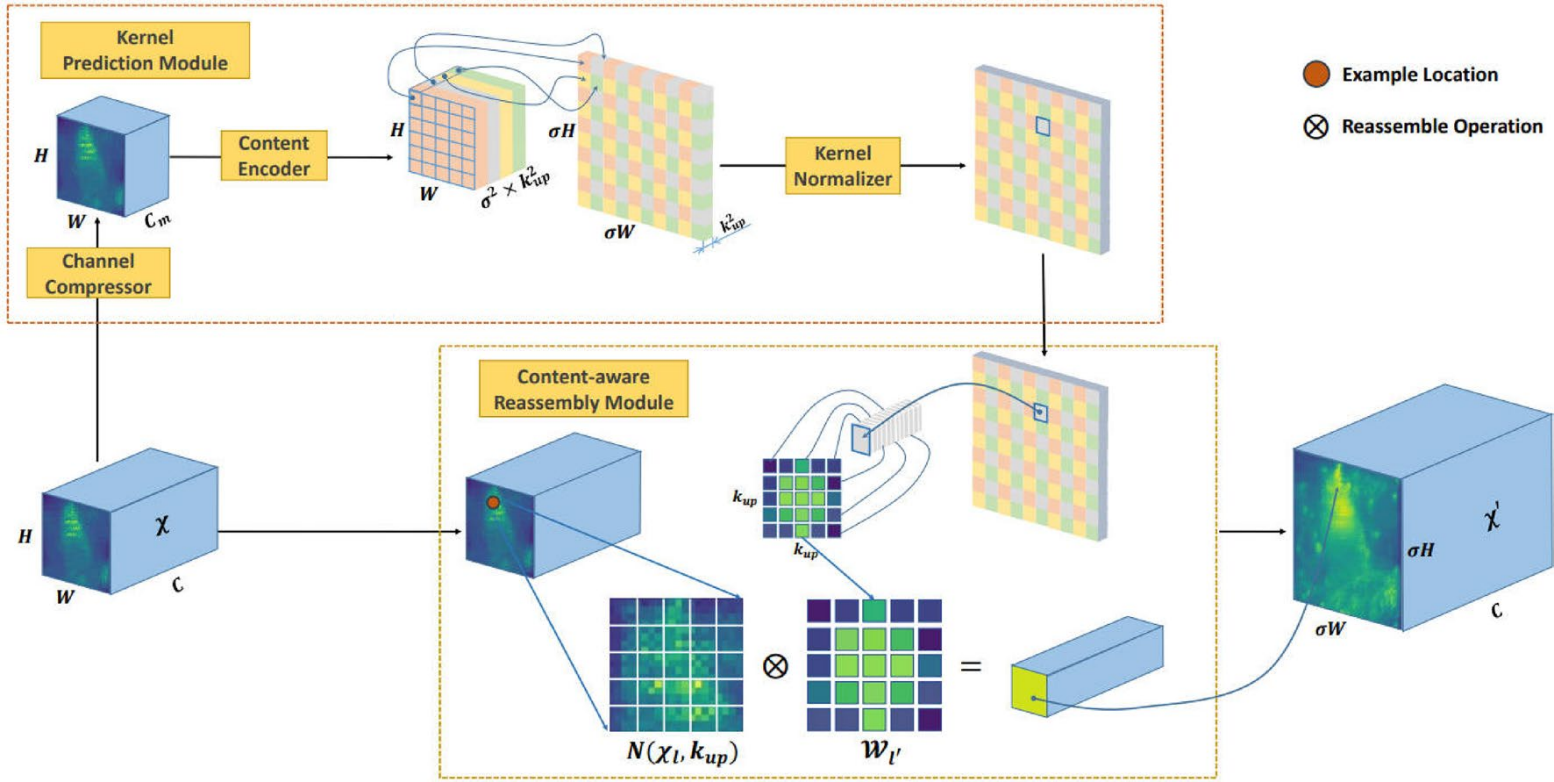
The overall framework of CARAFE. CARAFE is composed of two key components,kernel prediction module and content-aware reassembly module. A feature map with size $C \times H \times W$ is upsampled by a factor of $\sigma (= 2)$ in this figure.

In underwater target detection tasks, selecting an appropriate upsampling method is crucial for enhancing model performance. Traditional sampling methods such as nearest neighbor and bilinear interpolation only consider subpixel neighborhoods and fail to capture the rich semantic information required for intensive underwater prediction tasks. In this study, a lightweight and efficient Content Sensing Feature Recombination (CARAFE) upsampling operation is introduced,as illustrated in Fig. 7. CARAFE has the capability to aggregate contextual information over a large receptive field, addressing the limitation of traditional methods that only utilize local sub-pixel neighborhoods, which can lead to information loss. Additionally, CARAFE generates adaptive kernels based on instance-specific content, whereas traditional methods such as deconvolution use fixed kernels, lacking the flexibility to adapt to different instances. Furthermore, CARAFE is computationally more efficient in execution^20^. Therefore, replacing the traditional upsampling method in YOLOv8 with CARAFE can reduce computational complexity and more effectively extract rich semantic features, thereby significantly improving model performance.

The computation process of CARAFE involves two main steps: the first step is to predict and generate the recombination kernel $W_{\hat{l}}$ based on the content at the target position through the upsampling kernel prediction module $\psi$, as shown in Eq. (1); the second step is to recombine the features using the predicted kernel $W_{\hat{l}}$ through the content-aware reassembly module $\phi$, thereby achieving efficient upsampling, as shown in Eq. (2).

1 $$\begin{aligned} W_{\hat{l}}= & {} \psi (N(X_l, k_{\text {encoder}})) \end{aligned}$$

2 $$\begin{aligned} X_{\hat{l}}'= & {} \phi (N(X_l, k_{\text {up}}), W_{\hat{l}}) \end{aligned}$$

For example, given an input feature map size of $C \times H \times W$, assume an upsampling factor of $\sigma$. CARAFE first predicts the recombination kernel for the content at each target position. Then it completes the upsampling using adaptive and optimized recombination kernels. Finally, the output feature map size becomes $C \times \sigma H \times \sigma W$.

## Experiments

### Experimental details

Regarding the hardware environment, we used a 14 vCPU Intel (R) Xeon (R) Gold 6330 CPU @ 2.00GHz and NVIDIA GeForce RTX 3090 GPU with a graphics memory size of 24 GB. In terms of software environment, we have chosen CUDA 11.3, CUDNN 8.2.2, and Python 3.8 as the compiler. The hyperparameter settings of the model are shown in Table 1.

**Table 1:** Hyperparameter configuration.

Method	Configuration
Learning rate	0.01
Momentum	0.0005
Batch size	16
Optimizer	SGD
Image size	$640 \times 640$
Epochs	200

### Experimental dataset

This experimental study employed the URPC dataset and the Zhanjiang Underwater Target Detection Competition Dataset, which are elucidated individually as follows.

#### The URPC dataset

This study utilized the Underwater Robot Professional Competition 2021 (URPC2021) benchmark dataset, which comprises images captured from video frames recorded by underwater robots in natural settings. This dataset contains 8200 underwater images with box-level annotations^24^. The targets evaluated in the experiment include four categories of seafood: “holothuria,” “echinus,” “scallop,” and “starfish.” To create the experimental dataset, the images were randomly divided into training, validation, and test sets in a 7:1:2 ratio, resulting in 5,718 images for training, 868 for validation, and 1614 for testing.

#### Zhanjiang underwater target detection competition dataset

The present experiment incorporates the Zhanjiang Underwater Target Detection Competition Dataset as supplementary data, encompassing five categories: “holothuria,” “echinus,” “scallop,” and “starfish.” and “waterweeds”. This dataset comprises 5543 training images, where waterweeds is officially deemed negligible, accounting for only 82 targets. However, to assess the algorithm’s capability in detecting underwater small targets, waterweeds is still included in the detection category. Ultimately, this study employs 5543 images and five categories, partitioned randomly into training, validation, and testing sets at a ratio of 7:2:1^25^.

### Model evaluation metrics

This study employed metrics such as Precision, Recall, Intersection over Union (IoU), Mean Average Precision (mAP), and Frames Per Second (FPS) to evaluate the improved model.

IoU is calculated using Eq. (1) to quantify the overlap between the predicted bounding box and the actual bounding box. If the IoU of the detection result exceeds the threshold, it is true positive (TP), if it is below the threshold, it is false positive (FP), and if the undetected target is false negative (FN).

3 $$\begin{aligned} IoU = \frac{DetectionResult \cap GroundTruth}{DetectionResult \cup GroundTruth} \end{aligned}$$

Precision reflects the proportion of positive classes correctly classified by the model (Eq. 2). Recall indicates the proportion of positive classes correctly identified out of the total positive classes (Eq. 3). AP (Average Precision) is calculated based on Precision and Recall at different thresholds, with a larger area under the curve indicating higher recognition accuracy (Eq. 4).

4 $$\begin{aligned} Precision= & {} \frac{TP}{TP+FP}\end{aligned}$$

5 $$\begin{aligned} Recall= & {} \frac{TP}{TP+FN}\end{aligned}$$

6 $$\begin{aligned} AP= & {} \int _{0}^{1} P(r)dr \end{aligned}$$

mAP integrates the Precision and Recall across all categories, determined by calculating the area under the PR curve for each category, with higher values indicating better multi-category performance of the model (Eq. 5). Here, N represents the number of all classes, and indicates the average precision of the Nth class. FPS measures the processing speed of the model, with higher values indicating faster speed.

7 $$\begin{aligned} mAP=\frac{1}{N} \sum _{n=1}^{N}AP_{n} \end{aligned}$$

### Experimental results and analysis

#### Detailed performance analysis of the model

Given that the URPC dataset offers a broader spectrum of scenarios and target types, facilitating a more comprehensive evaluation of the model’s performance across diverse conditions. Therefore, subsequent experiments and analyses in this study will focus primarily on the URPC dataset.

**Figure 8:**
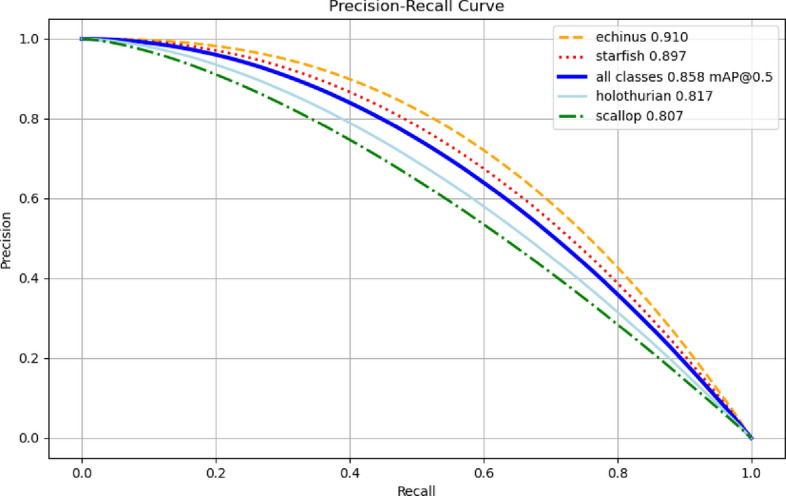
The precision-recall curve of the YOLOv8-LA model on the URPC dataset.

The utilization of the P-R curve stems from its effective illustration of the trade-off between accuracy and recall rate at varying thresholds. Accordingly, this study performed experiments to depict the recognition rates of different underwater organisms using the P-R curve by employing the YOLOv8-LA model. As shown in Fig. 8, the detection accuracy of the improved model was improved in all categories, especially for echinus, which reached 90.8%. After calculation, the average accuracy map of the model is 84.7%.

**Figure 9:**
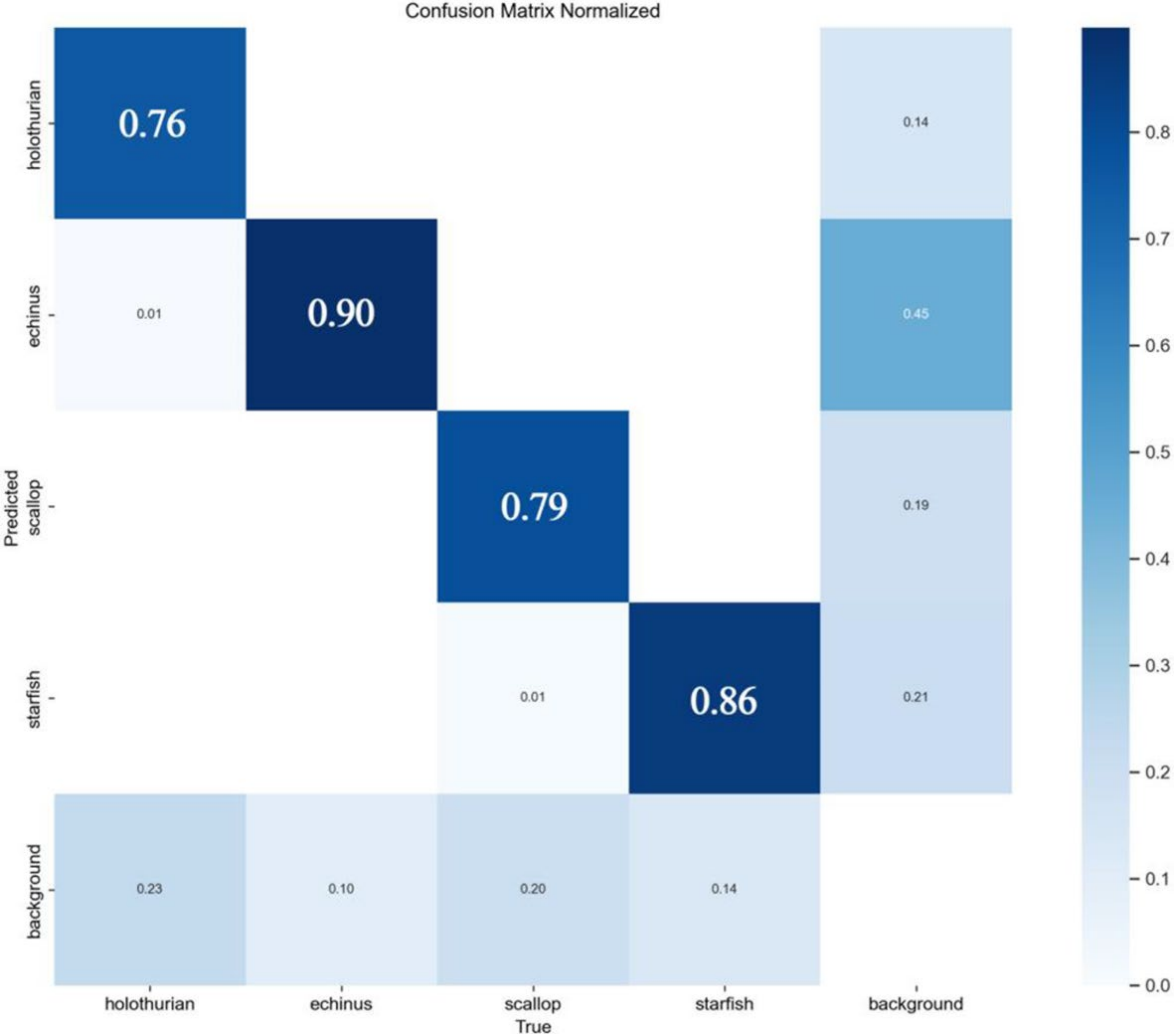
The confusion matrix of the YOLOv8-LA model on the URPC dataset.

Next, a confusion matrix was utilized to assess the accuracy of the YOLOv8-LA model’s predictions. In the confusion matrix, each column of the confusion matrix represents the predicted proportion of each category, while each row represents the true proportion of each category in the data, as shown in Fig. 9. The analysis of the confusion matrix shows that the prediction accuracy of “holothurian”, “echinus”, “‘scallop”, and “starfish” are 76%, 88%, 77%, and 86%, respectively, further confirming that the model has high accuracy in various categories.

**Figure 10:**
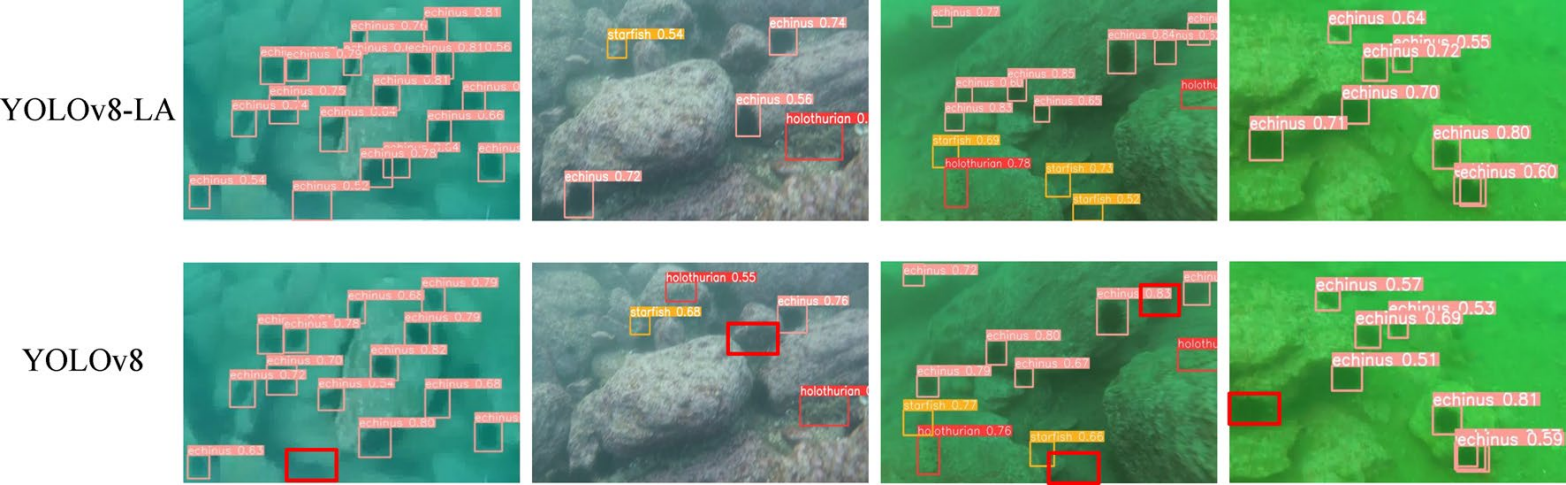
Detection results of YOLOv8-LA (first row) and YOLOv8 (second row) in adverse underwater scenarios. The red boxes indicate missed targets.

In addition, in order to more intuitively demonstrate the performance of the YOLOv8-LA model, we also conducted qualitative comparative analysis with YOLOv8. As shown in Fig. 10, YOLOv8-LA performs better than YOLOv8 in reducing error detection and missed detections, accurately detecting small-sized targets, and significantly improving its accuracy in predicting bounding boxes.

#### Ablation experiment

In this section, we explore the impact of various components and architectural modifications on the performance of YOLOv8 through ablation studies. Table 4 presents the variations in mean Average Precision (mAP) at a threshold of 0.5, frames per second (FPS), the number of parameters, and the computational cost measured in GFLOPS for each model configuration.

**Table 2:** Ablation comparison of model performance improvement on the URPC dataset.

Model	AP-FasterNet	LEPC	CARAFE^20^	mAP@0.5(%)	FPS	Parameters	GFLOPS
YOLOv8	$\times$	$\times$	$\times$	82.3	205.4	3,157,200	8.2
	$\checkmark$	$\times$	$\times$	84.4	150.2	2,565,916	7.4
	$\checkmark$	$\checkmark$	$\times$	84.1	**225**.**3**	2,482,256	**7**.**3**
	$\checkmark$	$\checkmark$	$\checkmark$	**84**.**7**	189.3	**2,422,688**	7.5

Bold text indicates the best result.

This ablation study evaluates the effects of incorporating various components into YOLOv8. The baseline YOLOv8 achieves an mAP@0.5 of 82.3%, 205.4 FPS, 3.16 M parameters, and 8.2 GFLOPS. With the addition of the AP-FasterNet module, the mAP improves to 84.4%. This improvement can be attributed to the enhanced feature extraction capabilities provided by AP-FasterNet, which captures more relevant features for detection. However, this results in a decrease in the frame rate to 150.2 FPS and a reduction in parameters to 2.57 M and 7.4 GFLOPS due to the additional computational overhead.Integrating the LEPC module maintains mAP at 84.1%, significantly increasing the frame rate to 225.3 FPS by optimizing parallel computation, and reducing parameters to 2.28 M and GFLOPS to 7.3. Introducing CARAFE enhances mAP to 84.7%, slightly reducing the frame rate to 189.3 FPS due to enhanced feature representation Table 2.

#### Comparative analysis of model performance on different datasets

We conducted a comprehensive comparison of the performance of the YOLOv8-LA model with other models, as shown in Table 3. On the URPC dataset, the YOLOv8-LA model proposed in this study exhibits superior performance in multiple key metrics,achieving an mAP@0.5 of 84.7% and an mAP@0.95 of 50.2%, both outperforming YOLOv8n, which scores 82.3% and 48.9% respectively.

The performance improvement can be attributed to several factors. The AP-FasterNet module enhances the feature extraction capability and improves the detection accuracy.The LEPC module optimizes the parallel computation and significantly improves the frame rate. In contrast, Faster R-CNN, as a two-stage detection algorithm, is known for its accuracy but slow processing speed, with a mAP of 74.3% and a frame rate of only 7.3 FPS. YOLOv8-LA not only outperforms Faster R-CNN in detection metrics, but also maintains a frame rate of 189.3 FPS, which is much higher than the requirement of real-time detection. This improvement is due to YOLOv8-LA’s single-stage detection architecture, which simplifies the detection process and increases speed without compromising accuracy.

Another noteworthy model is RTD-YOLOv5, which, despite its slightly higher accuracy of 84.3%, has a frame rate of only 6.3 FPS, suggesting that it sacrifices speed for slightly higher accuracy in its underwater detection processing. YOLOv8-LA, on the other hand, maintains a balance between high-speed processing and high accuracy. Other models such as YOLOv7, YOLOv5s variant and YOLOX-s also show competitiveness in some aspects, but their combined efficiency and effectiveness are not as good as YOLOv8-LA, which is mainly attributed to the synergistic optimization of the integrated modules, which achieves double optimization of the computational efficiency and detection accuracy.

**Table 3:** Performance comparison of object detection models on the URPC dataset.

Method	Precision (%)	Recall (%)	mAP@0.5 (%)	mAP@0.95 (%)	FPS	Parameters	GFLOPS
yolov5s^26^	83.1	76	82.4	46.5	125	7,074,330	N/A
yolov6^29^	N/A	N/A	83.2	83.2	N/A	N/A	N/A
yolov7^30^	81.6	75.2	82.4	47.2	57.80	36,547,292	103.3
YOLOv8n	82.6	76.3	82.3	48.9	**215.4**	3,157,200	8.2
Faster RCNN^31^	75.2	64.6	74.3	41.5	7.3	1,347,000,000	N/A
yolov5s+CBAM^32^	83.7	76.2	75.3	41.7	91	7,074,940	N/A
yolov5s+SAGHS^32^	78.1	73.7	79.2	45.1	125.3	7,074,330	N/A
YOLOX-s^33^	N/A	N/A	82.1	47.4	N/A	8,940,000	5.63
LUO-YOLOX^33^	N/A	N/A	80.7	46.7	N/A	5,310,000	**3.81**
RTD-YOLOv5^34^	84.3	72.8	82.4	45.9	6.3	5,900,000	N/A
**YOLOv8-LA**	**84.9**	**76.8**	**84.7**	**50.2**	189.3	**2,422,688**	7.5

Bold text indicates the best result.

In addition, to further validate the superiority of the proposed YOLOv8-LA model, we also evaluated commonly used underwater object detection models on the Zhanjiang Underwater Object Detection Competition dataset. As shown in Table 4, bold text represents the best result.

On the Zhanjiang dataset, YOLOv8-LA achieved superior performance with an mAP of 84.2%, a frame rate of 176.1 FPS, and an inference time of 4.92 ms. In contrast, Faster R-CNN, with an mAP of 81.88%, has a lower frame rate of 17.2 FPS and a high inference time of 58.12 ms due to its computationally intensive two-stage detection process. SSD, despite a high frame rate of 160.27 FPS, has a lower mAP of 79.25%. This lower accuracy is due to SSD’s simpler architecture, which trades off detection precision for higher speed. RetinaNet, with an mAP of 73.75% and a frame rate of 48.03 FPS, shows lower speed and accuracy, likely due to its reliance on Focal Loss, which, while improving performance on hard examples, increases computational complexity.MBFNet, while achieving high precision in detecting Holothurian (90.06%), exhibits an overall lower mAP of 82.29%, a frame rate of 48.94 FPS, and an inference time of 24.97 ms, highlighting its inefficiency in balancing computational speed and detection accuracy. These comparisons highlight YOLOv8-LA’s balanced and superior performance.

**Table 4:** Performance comparison of target detection model on the Zhanjiang dataset.

Method	AP(%)				mAP@0.5(%)	Param(M)	GFLOPs	FPS	Inf. Time(ms)
	Scallop	Starfish	Holothurian	Echinus					
Faster RCNN^31^	N/A	N/A	N/A	N/A	81.88	28.3	909.57	17.2	58.12
SSD^35^	N/A	N/A	N/A	N/A	79.25	3.941	**2.653**	160.27	6.22
CenterNet^36^	N/A	N/A	N/A	N/A	75.79	33.67	70.21	141.21	7.08
RetinaNet^37^	N/A	N/A	N/A	N/A	73.75	36.39	69.71	48.03	20.81
YOLOv3^38^	67.31	74.87	55.21	78.28	68.92	61.54	65.62	94.49	10.69
YOLOv4^39^	N/A	N/A	N/A	N/A	78.61	63.95	59.98	75.33	13.28
YOLOv5m	70.82	77.87	77.79	86.17	78.16	21.01	21.39	52.75	18.95
YOLOv5l	76.17	79.17	73.60	88.3	79.13	46.65	48.42	90.31	11.07
YOLOx^40^	65.28	76.68	51.58	84.4	69.49	54.15	65.77	43.95	22.75
YOLOv7^30^	81.7	**86.8**	91.0	74.6	81.48	37.62	103.2	49.49	20.21
DETR^41^	69.93	84.28	62.07	87.22	75.87	36.74	31.92	46.28	21.64
YOLOv4-AFFM^42^	73.06	86.00	66.85	90.14	79.01	10.73	63.22	44.18	21.47
YOLOx-DCA^43^	79.22	86.77	72.28	88.73	81.75	4.51	25.35	56.73	27.28
YOLOv5m-PPM	66.62	80.08	51.98	84.53	70.08	N/A	N/A	N/A	N/A
YOLOv5l-PPM	71.89	83.31	59.19	86.19	75.14	N/A	N/A	N/A	N/A
DETR-PPM	78.51	86.55	71.39	87.91	81.09	N/A	N/A	N/A	N/A
MBFNet^15^	80.93	81.36	76.74	**90.06**	82.29	26.63	48.94	40.05	24.97
**YOLOv8-LA**	**87.10**	85.90	**91.50**	82.10	**84.20**	**2.27**	6.10	**176.1**	**4.92**

Bold text indicates the best result.

## Conclusion

In this paper, we introduce YOLOv8-LA, a novel network designed specifically for underwater object detection tasks. Our approach integrates the Lightweight Efficient Partial Convolution (LEPC) module and the AP-FasterNet module to enhance both detection accuracy and computational efficiency. The LEPC module, replacing the traditional C2F module, reduces the parameter count and improves computational speed through selective convolution, while the AP-FasterNet module, incorporated for the first time, replaces the feature extraction module of YOLOv8, thereby improving the accuracy of small object detection and enhancing network stability.Our experiments demonstrate that YOLOv8-LA significantly outperforms mainstream methods in various metrics, particularly in challenging underwater environments, showcasing its robustness and real-time detection capabilities. The integration of these modules has effectively improved the accuracy and speed, establishing the strong competitiveness of YOLOv8-LA.

However,despite these advancements, YOLOv8-LA encounters limitations due to the increased feature extraction process,which leads to a reduction in speed. In future work, we aim to explore additional factors affecting the feature extraction process and investigate more advanced network architectures to further optimize the network’s performance. This will facilitate better deployment on mobile platforms and extend its applications to other downstream tasks to demonstrate the broad applicability of the network.

## Data Availability

The dataset of Zhanjiang competition used in this study are publicly available at https://github.com/BIGWangYuDong/lqit. The dataset of URPC2021 used in this study are publicly available at https://aistudio.baidu.com/datasetdetail/80480/0.

## Code availability

The source code will be made publicly available at https://github.com/zzuilcc/YOLOv8-LA.git.
